# Accuracy of a Rapid and Non-Invasive Method for the Assessment of Small Fiber Neuropathy Based on Measurement of Electrochemical Skin Conductances

**DOI:** 10.3389/fendo.2016.00018

**Published:** 2016-02-29

**Authors:** Lyse Bordier, Manuel Dolz, Linsay Monteiro, Marie-Laure Névoret, Jean-Henri Calvet, Bernard Bauduceau

**Affiliations:** ^1^Service d’Endocrinologie, Hôpital d’Instruction-des-Armées-Bégin, Saint-Mandé, France; ^2^Impeto Medical, Paris, France

**Keywords:** small fiber neuropathy, accuracy, reproducibility, sudomotor function, sweat glands

## Introduction

Peripheral nerves (PN) consist of small and large fibers (1). The small fibers represent 80% of PN and are long, thin, with little or no myelin. They are, therefore, more fragile and the first to be damaged in many pathological processes (2–5). The current clinical diagnostic methods mainly assess large fibers (6). Similarly the gold standard neurophysiological tool, namely nerve conduction studies or NCS, is also limited to measuring large fiber function. These recommended methodologies, therefore, only examine 20% of PN, those that are largest and degenerate late or not at all in certain diseases.

Small fibers can be sensory or autonomic and there are several methods to assess small fiber function or structure (7). Laser-evoked potentials assess A-delta fiber function (sensory nerves) but these instruments are not widely available (8). Quantitative sensory testing (QST) measures sensitivity to cold, heat, and vibration (sensory small and large fibers) and is more widely available but time-consuming and subjective for routine clinical practice (9). Skin biopsies can assess small fiber structure but are relatively invasive (3 mm in diameter, 1 month for full healing) and, thus, are ill suited for longitudinal assessments (10).

Sudomotor function assessing small fibers of the sympathetic autonomic system can be evaluated by the quantitative sudomotor axon reflex test (QSART); though considered the reference method, QSART remains mostly limited to research centers due to the technical complexity and relative discomfort of the examination (11). Other sudomotor methodologies include the Neuropad, which is semi-quantitative and not highly sensitive (12), quantitative direct and indirect testing (QDIRT) and the dynamic sweat test (DST). QDIRT and DST both induce sweating with iontophoresis of acetylcholine or pilocarpine; they are relatively tedious and not particularly suited to the outpatient clinic and there are no data validating the diagnostic utility of these newer technologies (13, 14). Specific stains can be used to evaluate sweat gland nerve fiber density (SGNFD), but there is currently no standardized methodology or normative reference ranges for SGNFD (15).

The SUDOSCAN^®^ device was developed to allow the quantitative measurement of sweat gland function using a simple and rapid process (16, 17). Results are immediately available and expressed as electrochemical skin conductances (ESCs). This technique has been compared to reference neurological tests, is not operator dependent, and could be used in the follow-up of patients and in multi-center studies (18–21).

The aim of this study was to assess repeatability and reproducibility of the method in healthy volunteers (HV) and diabetic patients with a range of glycemic control.

## Methods

The study was performed in Bégin Hospital, Saint-Mandé, France on 18 HVs and 14 patients with type 2 diabetes (PTD2). All participants gave their written informed consent.

SUDOSCAN^®^ (Impeto Medical, Paris, France) is a patented device designed to perform a quantitative evaluation of sweat gland function based on the electrochemical reaction between ions in sweat (mainly chlorides at the anode and protons at the cathode) and stainless-steel electrodes. The apparatus consists of two sets of electrodes in contact with the palms of the hands and soles of the feet, where sweat gland density is highest, connected to a computer for recording and data management purposes [see depiction in Ref. (21)]. To conduct the test, the individual is required to stand still for 2 min. During the test, 4 combinations of 15 different low direct current (DC) incremental voltages ≤4 V are applied and electrodes serve alternatively as anode or cathode. Neither special subject preparation nor specially trained medical personnel is required to complete the test. ESC in the hands and feet, i.e., the ratio between current generated and the constant DC stimulus, are displayed on a monitor immediately after the test.

The principle of the method, including an electrochemical skin model, has been partially published and establishes that the concentrations of the electro-active species (i.e., chlorides near the anode, protons near the cathode) and the electric potential are mostly constant inside the sweat gland (22). The measured ESC equals the electric permeability of the gland wall against the electro-active species. In the human skin, including hairless skin such as the palms of the hands and soles of the feet, the stratum corneum is electrically insulating against DC voltages under 10 V and only the appendageal pathways of the eccrine sweat glands are conductive (23). Thus, ESC depends neither on the thickness of the stratum corneum nor on the sweat composition or conductivity, hence favoring a good reproducibility.

Sweat glands are innervated by the sympathetic autonomic peripheral nervous system with 70% cholinergic-muscarinic pathway and 30% adrenergic pathway (24). The physiological process of sweating starts with a “chemical” stimulus, for example, in the cholinergic pathway, the binding of acetylcholine to muscarinic neuroreceptors that will modulate ion channels, creating a flux of ions through the membrane polarizing the gland to voltages around 10 mV. In the ESC technology, measurement is based on an initial electrical stimulus: it directly polarizes the gland with voltages between 100 and 1000 mV. This induces ion fluxes across the gland wall, depending on the electrochemical gradient of the ions. Because the current applied is high compared to the physiological current involved in ion exchange, the test can be considered a “stress test” for sweat glands.

Two measurements on three different Sudoscan devices were performed under usual testing conditions on each HV and PTD2. Measurements were performed in the same order for all subjects: first measurement on devices 1, 2, and 3 and then second measurement on devices 3, 2, and 1. Electrodes were cleaned after each test and an interval of at least 5 min was observed between two successive measurements.

A blind analysis of the data was performed by an independent party.

### Statistical Analyses

Comparison of measures recorded at different times on the three different devices was performed according to the ISO 5725-2 standard “Accuracy (trueness and precision) of measurement methods and results – Part 2: Basic method for the determination of repeatability and reproducibility of a standard measurement method” (25).

Results for quantitative variables are shown as means ± SD. The data management and statistical analysis were done using SAS version 9.4 and R version 3.2.1. Bland–Altman analysis was used to assess agreement and bias between two measurements performed on the same device. Intraclass correlation coefficient (ICC) was calculated with the mean values obtained on each device to compare reproducibility between the three devices.

## Results

The databases for this Report have been deposited in a public repository (figshare). Access is provided using the following link: http://figshare.com/s/f7792f2093c411e5b7e706ec4bbcf141

– Demographics.xlsx contains the demographic information of the healthy controls and diabetic patients, with each individual denoted as the letter “S” followed by an Arabic numeral;
– Reproducibility_healthy controls.xlsx contains the Sudoscan ESCs recorded for each subject for the hands (first sheet) and the feet (second sheet). The repeatability and reproducibility analyses are compiled for the hands ESC on the third sheet and for the feet ESC on the fourth sheet.
– Reproducibility_diabetic subjects.xlsx contains the Sudoscan ESCs recorded for each subject for the hands (first sheet) and the feet (second sheet). The repeatability and reproducibility analyses are compiled for the hands ESC on the third sheet and for the feet ESC on the fourth sheet.

Participants were recruited and tested in September–October 2015. Demographic characteristics of the HV were mean age: 37 ± 13 years, mean BMI: 26 ± 4, 72% males. Among the PDT2 patients, these were mean age: 62 ± 9 years, mean BMI: 29 ± 5 kg/m^2^, 71% males, mean HbA_1C_: 7.0 ± 0.9%, and mean diabetes duration: 10.8 years (range 5.5–15.0). All PDT2 had documentation of vibration perception, 10-g monofilament testing and ankle reflexes: eight patients had all normal results, four had one abnormal clinical sign, and two had two or more abnormal results.

Mean ESCs for all the measurements performed were 75.8 ± 7.3 μS in HV and 62.6 ± 10.4 μS in PDT2 for the hands and 75.4 ± 5.5 μS in HV and 69.2 ± 9.4 μS in PDT2 for the feet. For hands ESC, the mean repeatability SD was 3.1 μS (mean coefficient of variation was 4.2 ± 2.7%) and the mean reproducibility SD was 3.2 μS (mean coefficient of variation was 4.3 ± 2.7%) in HV, while they were 4.3 μS (mean coefficient of variation was 7.1 ± 5.9%) and 4.5 μS (mean coefficient of variation was 7.4 ± 6.1%), respectively, in PT2D. For feet ESC, the mean repeatability SD was 2.1 μS (mean coefficient of variation was 2.8 ± 1.6%) and the mean reproducibility SD was 2.3 μS (mean coefficient of variation was 3.1 ± 1.5%) in HV, while they were 4.3 μS (mean coefficient of variation was 6.9 ± 6.3%) and 4.3 μS (mean coefficient of variation was 6.9 ± 6.3%), respectively, in PT2D.

**Figure 1:**
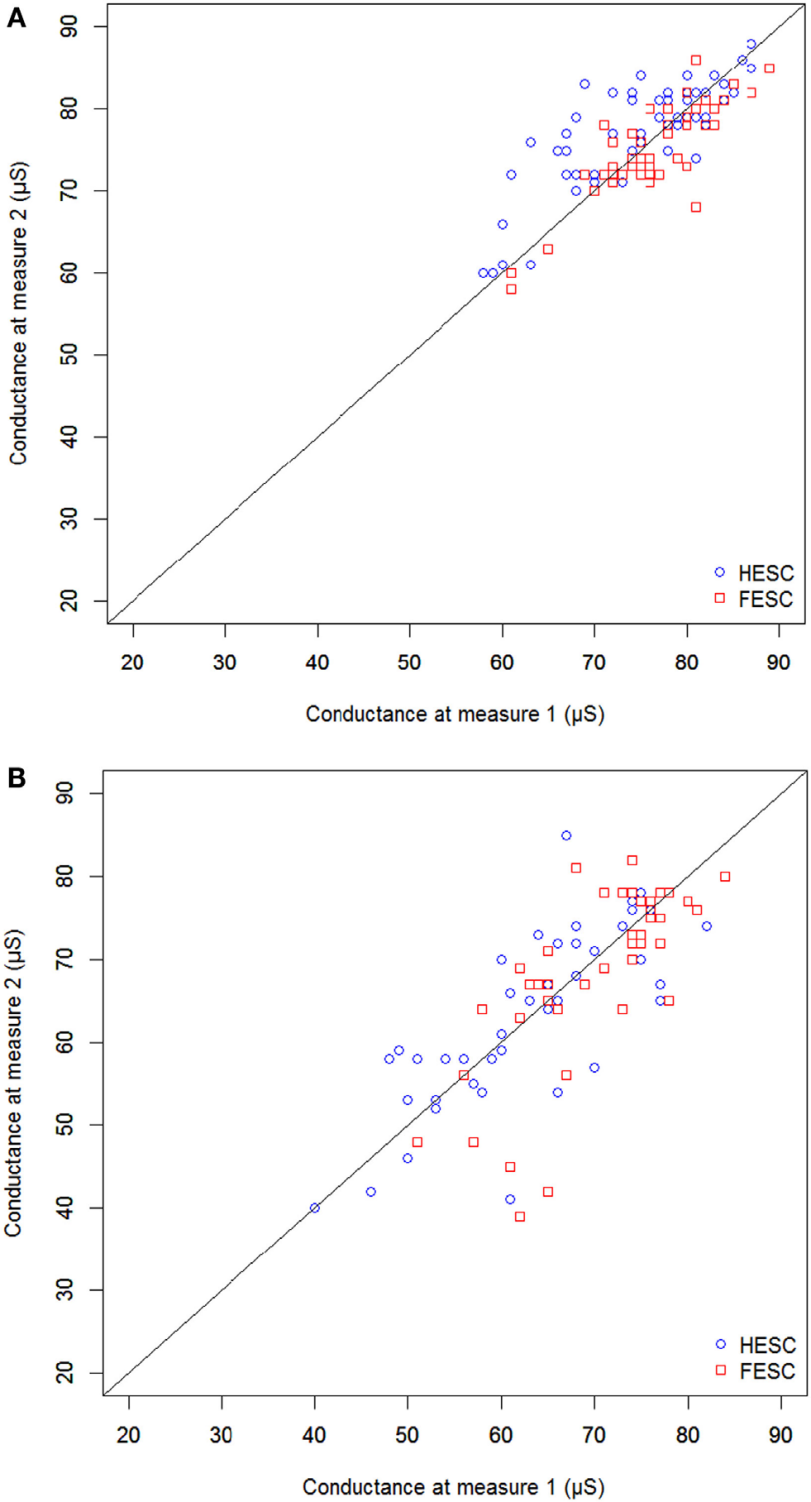
**Correlation between two successive measurements performed with the same device [(A) healthy population and (B) patients with type 2 diabetes]**.

**Figure 2:**
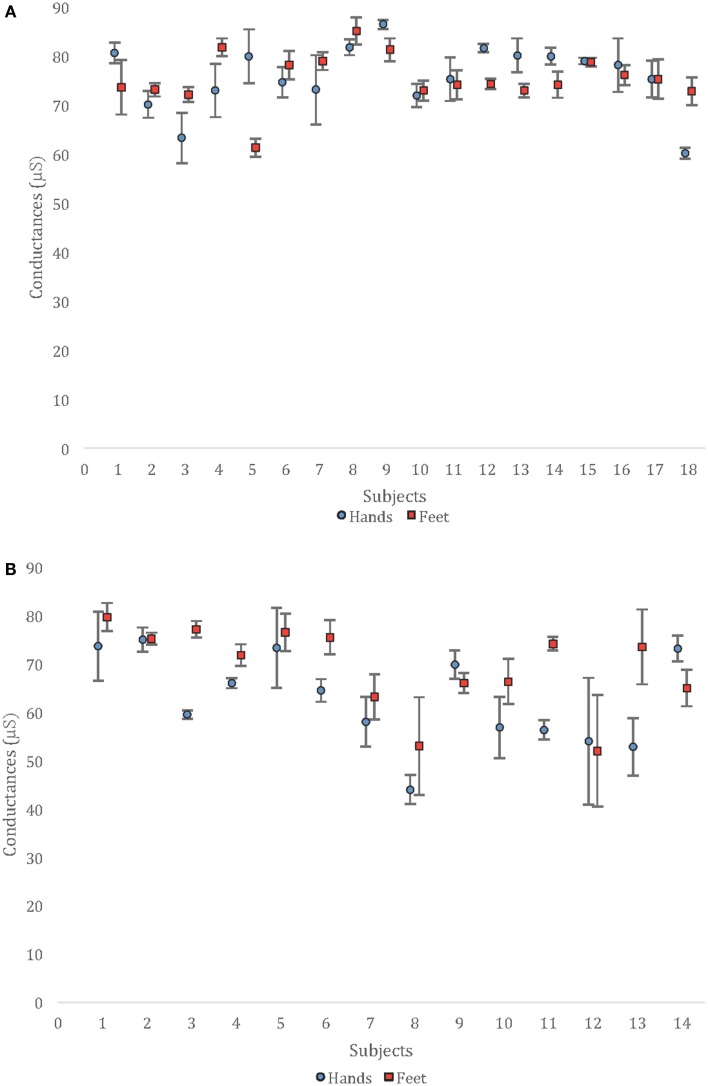
**Mean values and SD observed for the six measurements of each subject [(A) healthy population and (B) patients with type 2 diabetes]**.

Repeatability is illustrated in Figure 1; reproducibility is depicted in Figure 2 with mean ± SD of all the measurements performed on the three devices. ICC used to compare the three devices were 0.87 (0.74–0.94) and 0.85 (0.71–0.93) in HV and 0.95 (0.89–0.98) and 0.88 (0.74–0.96) in PDT2 for feet and hands, respectively.

## Discussion

This comparison between tests performed twice on three different devices demonstrates that for feet ESC the mean coefficient of variation for repeatability was 2.8 ± 1.6% in HV and 6.9 ± 6.3% in PDT2, while coefficient of variation for reproducibility were 3.1 ± 1.5 and 6.9 ± 6.3%, respectively. Similar or slightly higher values were observed for hands ESC.

Coefficients of repeatability and reproducibility are important for a test method to be useful in the follow-up of patients or in multi-center studies; differences in test results over time can be expected to reflect a true physiological change rather than the margin of error of the test itself. ESC repeatability and reproducibility were previously assessed in several studies but never according to an accepted international standard, i.e., six measurements for each subject in the same study. This accuracy study was eminently feasible due to the test’s (i) rapidity and ease of use, (ii) good acceptance by the patients, and (iii) lack of susceptibility to habituation, unlike some sudomotor function tests (26). The more common methods used in the assessment of peripheral neuropathy would not have allowed for such complete accuracy testing.

The results obtained in this study are in accordance or even better than previously reported coefficients of variation for ESC measurements (18, 27). The higher variation observed for hands ESC compared to feet ESC mainly in HV remain acceptable for measurements performed according to usual practice. In fact, it is most likely explained by the difference in the contact of the hands on the electrodes: whereas the feet are aided by gravity to maintain constant pressure on the electrodes throughout the test, the palms must be consciously maintained flat and steady for optimal test quality. The mean hands and feet ESCs observed among PDT2 were lower than those in HV, most likely secondary to diabetic peripheral neuropathy in some of the patients. However, the coefficients of repeatability and reproducibility remained acceptable.

Statistical tests used to compare measurements were issued from ISO 5725-2 standard “Accuracy (trueness and precision) of measurement methods and results – Part 2: Basic method for the determination of repeatability and reproducibility of a standard measurement method” as recommended by Food and Drug Administration and completed with Bland–Altman analysis and ICC that are more frequently reported in publications. All analyses demonstrated repeatability and reproducibility values that are quite acceptable and comparable to variations observed with other tests used to assess peripheral neuropathies.

Several factors may explain the good repeatability and reproducibility of the ESC measurement:

(a) There is a direct and calibrated electrical stimulation of the sweat gland, contrary to methods that depend on a pharmacological stimulus that is potentially more variable (28).
(b) The stratum corneum is electrically insulating against DC voltages under 10 V and only the appendageal pathways of the eccrine sweat glands are conductive; thus, ESC does not depend on the thickness of the stratum corneum (23).
(c) The electrode is firmly applied to the skin, effectively plugging the pores of the glands and blocking any physiological sweat flow, thus minimizing a possible dependence of the test on conditions that could increase this flow, such as high temperature or exercise.
(d) The stainless-steel electrodes are sensitive to chlorides, a key factor for the electrochemical reaction (29).
(e) The active measurement is conducted with the same constant polarizations at voltage levels that are at least 10 times higher than physiological ones; hence, ESC does not depend on the sweat composition or conductivity.
(f) ESC is computed by the slope of the curve (resulting current – applied voltage) and, therefore, is not affected by any electrochemical over potential of the electrode (30).
(g) The internal electronic circuit of the technology can measure the voltages precisely, with an accurate analog-to-digital converter having a resolution of 10 bits.

Results observed in this study can be compared to other methods used for assessment of small or large fiber structure or function. The variability for skin biopsy was investigated by Smith et al. on IENFD measurements from different sections and punches made by two different observers (inter-observer variability) using a total of 48 punch biopsies obtained from 22 patients. Intra-observer variability (two measures by the same observer) was also determined for 50% of the sections and punches. Mean (SD) inter-observer variability was 9.6 ± 9.4% for each biopsy site and 10.2 ± 11.9% for individual sections. Mean intra-observer variability was 9.6 ± 8.9% for biopsies, and 8.8 ± 9.0% for sections (31).

Repeatability of QSART and QST was assessed on 23 patients: 19 patients underwent repeat QST testing, and 13 patients underwent repeat QSART testing. QST (cold detection threshold ICC was 0.80, vibration detection threshold ICC was 0.75) was more reproducible than QSART (ICC foot 0.52). Repeatability of QSART was similar for the forearm, proximal leg and foot sites (0.52–0.63). The distal leg site was the least repeatable, with an ICC of 0.42. The rather poor repeatability of QSART could be explained by the fact that the authors used equipment similar but not identical to the original device designed at the Mayo Clinic (32). Sletten et al. established that the current delivered for acetylcholine iontophoresis i.e., the stimulus for the axon-reflex sweat production depends on the characteristics of the stimulator, return electrodes, and the skin resistance of the participant and is a critical issue for test performance (28).

Smith et al. determined the reproducibility of corneal confocal microscopy, a method to assess small fiber neuropathy in the cornea of 11 normal subjects using the same device (a Heidelberg Retinal Tomography III microscope) in five standardized locations. Nerve fiber length showed high reproducibility, with a relative inter-trial variability (RIV) of 5.02 ± 2.8% and ICC of 0.92 when five locations were averaged, and 6.92 ± 5.3% and ICC of 0.84 when four were averaged. Tortuosity coefficient was less reproducible, with a mean RIV of 13.6 ± 11% and ICC of 0.57 (*p* < 0.030) when five images were averaged (33).

Papanas et al. investigated reproducibility of Neuropad and showed that in patients with sudomotor dysfunction, intra-observer CV ranged between 4.2 and 5.1% (right foot) and between 4.1 and 5% (left foot), while inter-observer CV ranged between 4.3 and 4.9% (for either right or left foot). In patients without sudomotor dysfunction, intra-observer CV ranged between 4.1 and 4.8% (right foot) and between 4.1 and 4.7% (left foot), while inter-observer CV ranged between 4.3 and 4.7% (right foot) and between 4.2 and 4.5% (left foot) (34). No formal reproducibility of QDIRT or DST could be identified in the English language literature.

Taksande et al. computed the inter-observer reproducibility between two examiners for the diagnosis of peripheral neuropathy in patients with type 2 diabetes using a clinical examination, including vibration perception, 10-g monofilament touch sensation, and ankle reflexes (35). Measures of reproducibility used were percent agreement and kappa statistic (percent agreement beyond chance). For impaired vibration, impaired sensation and absence of reflex, percent agreements were 83, 82, and 77, respectively, while kappas were 0.35 (0.11–0.60), 0.53 (0.35–0.72), and 0.45 (0.27–0.64), signaling moderate-to-poor reproducibility. Similarly, Maser et al. evaluated inter-observer variation in a neurological examination to assess small and large fiber function on three separate occasions. Among five non-diabetic subjects, the mean coefficients of variation (CV) of log10-transformed variables were for vibration testing: finger 20% and great toe 16%, and for thermal testing: finger 34% and great toe 24%; among five diabetic subjects, the mean CV were for vibration testing: finger 8% and great toe 8%, and for thermal test: finger 29% and great toe 26% (36).

There are some limitations to this analysis and ESC measurements: (i) sudomotor function is principally controlled by cholinergic activity; the effect of drugs with anti-cholinergic activity on the device accuracy has not been investigated; (ii) the diabetic subjects in this report are not fully characterized according to AAN polyneuropathy criteria (37); it is, therefore, not possible to ascertain whether these accuracy measures apply to all degrees of peripheral neuropathy; (iii) almost all individuals can safely undergo ESC testing; however, measurements cannot be obtained on individuals with an amputated hand or foot.

## Conclusion

This study establishes that repeatability and reproducibility of ESC measurements appear to be respectable in HVs and patients with type 2 diabetes. Variation is comparable to or lower than methods commonly used in the assessment of small or large fiber neuropathy especially those that are operator- or patient dependent. Based on these results and the ease, speed, and non-invasiveness of testing, the ESC method could be considered for the follow-up of patients and as an endpoint in multi-center trials.

## Author Contributions

LB, MD, and BB recruited study participants, executed the study protocol, compiled study data, contributed to authoring the manuscript, and provided final approval to the manuscript. LM and J-HC conceived the study protocol. LM performed all the statistical analyses and prepared manuscript figures. J-HC and M-LN drafted and revised the manuscript.

## Conflict of Interest Statement

LM, M-LN, and J-HC are employees of Impeto Medical. LB, MD, and BB have no conflict of interest.
